# Prediction of Crack Initiation Based on Energy Storage Rate during Low-Cycle Fatigue of Austenitic Stainless Steel

**DOI:** 10.3390/ma14195526

**Published:** 2021-09-24

**Authors:** Wojciech Grodzki, Wiera Oliferuk, Michał Doroszko, Jarosław Szusta, Leszek Urbański

**Affiliations:** 1 Department of Mechanics and Applied Computer Science, Faculty of Mechanical Engineering, Bialystok University of Technology, Wiejska 45A, 15-351 Bialystok, Poland; m.doroszko@pb.edu.pl (M.D.); j.szusta@pb.edu.pl (J.S.); 2 Institute of Fundamental Technological Research, Polish Academy of Sciences, Pawińskiego St. 5B, 02-106 Warsaw, Poland; wolif@ippt.gov.pl (W.O.); lurban@ippt.pan.pl (L.U.)

**Keywords:** low-cycle fatigue, infrared thermography, plastic strain localization, digital image correlation, energy storage rate, plastic instability criterion

## Abstract

The low-cycle deformation of 304L austenitic stainless steel was examined in terms of energy conversion. Specimens were subjected to cyclic loading at the frequency of 2 Hz. The loading process was carried out in a hybrid strain–stress manner. In each cycle, the increase in elongation of the gauge part of the specimen was constant. During experimental procedures, infrared and visible-range images of strain and temperature fields were recorded simultaneously using infrared thermography (IR) and digital image correlation (DIC) systems. On the basis of the obtained test results, the energy storage rate, defined as the ratio of the stored energy increment to the plastic work increment, was calculated and expressed in reference to selected sections of the specimen. It was shown that, before the specimen fracture in a specific area, the energy storage rate is equal to zero (the material loses the ability to store energy), and the energy stored during the deformation process is released and dissipated as heat. Negative and close-to-zero values of the energy storage rate can be used as a plastic instability criterion on the macroscale. Thus, the loss of energy storage ability by a deformed material can be treated as an indicator of fatigue crack initiation.

## 1. Introduction

Determining the fatigue properties of a material subjected to a cyclic strength test requires expensive research and time-consuming evaluation of obtained data. Considering that the deformation process causes an increase in temperature on the surface of the tested specimen, experimental approaches based on temperature measurement have been developed to provide reliable fatigue properties of tested material [1,2]. The thermal approach often leads to questionable results, because temperature variations depend on the thermal properties of tested materials and applied boundary conditions. Therefore, the physical meaning of the critical stress value obtained by thermal methods is not clear [3,4]. Further studies related to the determination and analysis of heat source dissipation in materials subjected to low-cycle fatigue [4,5,6,7] have led to the assumption that the dependency of dissipated energy in the form of heat with a cyclic response is not clear, whereas the stress–strain response during a fatigue test has a strict reference to the energy storage process [7]. This conclusion refers to the change in the material microstructure resulting in crack initiation and its impact on the energy storage process. Knowledge of the mechanical energy *w_p_* conversion into energies stored *e_s_* and dissipated as heat *q_d_* during the deformation process is an important aspect in characterizing material fatigue properties. The identification of energy transformation allows defining the instability of plastic deformation according to the principles of thermodynamics [8]. The instability of plastic deformation during the fatigue process undoubtedly leads to the initiation of fatigue fracture.

The ratio of the stored energy to the mechanical energy used in plastic deformation depends on strain state of the tested material [9,10,11,12,13,14,15,16]. Considering the above, the energy conversation measurement was conceptualized on the basis of the energy storage rate evaluation in each state of plastic deformation [17,18,19,20]. The energy storage rate *Z* is defined as the derivative of *e_s_* with respect to monotonically increasing *w_p_*, i.e.,

(1) $$Z=\frac{de_{s}}{dw_{p}},$$

or the ratio of finite increments Δ*e_s_* and Δ*w_p_*. Taking into account that

(2) $$\Delta e_{s}=\Delta w_{p}-\Delta q_{d},$$

the energy storage rate can be defined as

(3) $$Z=\frac{\Delta e_{s}}{\Delta w_{p}}=1-\frac{\Delta q_{d}}{\Delta w_{p}},$$

where the increments of stored energy *e_s_*, mechanical energy used in plastic deformation *w_p_*, and part of the mechanical energy expended on plastic deformation converted into heat *q_d_* determined in the presented work are expressed per unit of mass of the tested material.

A key feature in fatigue prediction is crack initiation. Damage accumulation during cyclical deformation of a material affects its microstructure, which results in the formation of cracks [21,22]. As already mentioned, these changes determine the process of energy storage in this material. In the previous work by Oliferuk et al. [20,23,24], it was shown experimentally that, before the fracture of a specimen subjected to monotonic uniaxial tension, the material reaches a state in which the energy storage rate value is equal to zero. This is equivalent to a loss of the material’s ability to store energy. Although mechanical energy in the form of deformation is delivered to the specimen, its internal energy does not increase. The loss of ability to store energy occurs in the area of plastic strain localization. An evaluation of the plastic instability criterion with the energy conversation principle can be achieved via measurement of the of energy storage rate value. The purpose of the present work is to examine whether, in a specimen subjected to low-cycle loading, the value of energy storage rate reaches 0. A positive answer to this question can allow concluding that the number of cycles after which the material loses its ability to store energy (*Z* = 0) can be treated as an indicator of fatigue crack initiation. The aim of the presented study is to determine the dependency of the energy storage rate on the number of cycles in the area of plastic strain localization, in order to predict fatigue crack initiation as a function of energy conversion. According to the literature analysis, no attempts have been made to predict fatigue crack initiation as a function of energy conversion in a material subjected to cyclic loading. Therefore, this paper presents a new approach to search for the macroscopic indicator of fatigue crack initiation during the low-cycle fatigue process. This indicator has a physical basis, because it results from the energy conversion during low-cycle fatigue of the tested material, while the energy conversion is a macroscopic reflection of changes in the microstructure of this material. An indicator of fatigue crack initiation may be the number of cycles at which the energy storage rate *Z* is equal to 0.

The experiments described in the present work were performed on austenitic stainless steel due to the possibility of obtaining high values of deformation and temperature before specimen fracture [25]. The considered material is used for parts of various installations (cooling and ventilation systems), environmental chambers, containers in civil construction, and mechanical components with requirements of corrosion resistance. The wide application range of stainless steel requires the determination of new techniques describing its fatigue properties. Considering a new research approach based on the measurement of temperature and strain fields, the selected material was tested in conditions of low-cycle fatigue.

## 2. Materials and Methods

Specimens were made of a 4 mm thin sheet of austenitic stainless steel, with the cutting orientation based on the cold-rolling direction of the material. Table 1 presents the chemical composition of the 304L steel (according to AISI). It is important to highlight that, during the preparation of samples, no material phase transformation occurred. It was verified that, after the applied treatments, the tested steel did not show magnetic properties.

**Figure 1:**
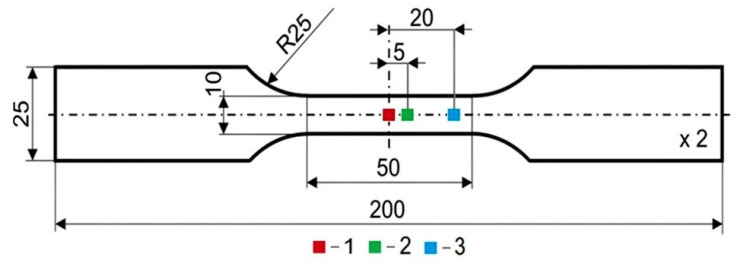
Tested specimen.

Specimen preparation consisted of five main stages: preliminary annealing, rolling, cutting, final annealing, and polishing. Steel strips with a 25 mm × 4 mm cross-section were initially annealed at 1050 °C and water-quenched. The annealing temperature was chosen by trial and error, to effectively reduce the texture of the rolled material. The strips were water-quenched to prevent the occurrence of carbides at the grain boundaries. The next step involved a 50% cold-rolling process of the strips to reduce their thickness to 2 mm. The obtained material was cut into the designed specimen geometry using the electro-erosion machining technique (Figure 1).

**Figure 2:**
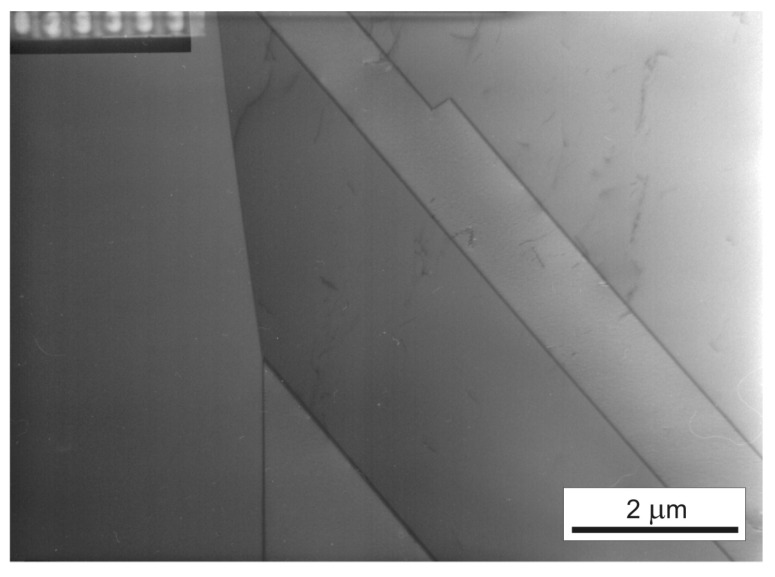
Typical microstructure of the initial state of the tested steel (TEM).

**Figure 3:**
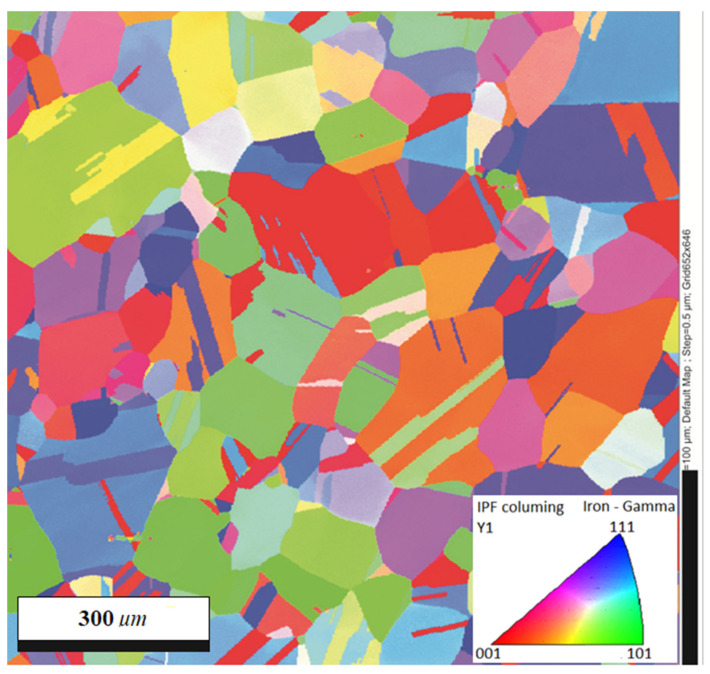
Distribution of grain orientation in the tested specimens (EBSD).

The prepared specimens were annealed in two stages: (1) at 950 °C for 20 min to remove the residual stress resulting from the production process, and (2) at 1180 °C for 150 min to obtain a homogeneous microstructure with a mean grain size of approximately 90 μm. In order to remove the texture resulting from the applied treatments and smoothing of the specimen surface, the electro-polishing technique was used. Optical metallographic (Zeiss, Oberkochen, Germany) and transmission electron microscopy (TEM, Zeiss, Oberkochen, Germany) observations of the tested material indicated complete recrystallization (Figure 2). The density of dislocations in the unloaded specimen was low and randomly distributed in the grain boundaries and matrix. Figure 3 presents the distribution of grain orientation in the tested specimens using electron backscatter diffraction microscopy (EBSD, EDAX, Mahwah, NJ, USA). The obtained results showed randomly oriented grains in the specimen.

In order to use a digital image correlation system for the determination of the displacement field during the deformation process, on one side of the specimen, a specific pattern of white and black areas was painted. The other side of the specimen was used for temperature field examination; therefore, this side of the sample was covered in a graphite conductive coating to ensure high and uniform emissivity of the surface (~0.95). The graphite coating was applied using an airbrush and a graphite suspension in water. Three different sections (marked 1, 2, and 3) of the specimen surface were selected for further analysis. Their location is shown in Figure 1. The cross-sectional area of the specimen was equal to 20 mm^2^. The volume of each selected section was equal to 2 mm^3^.

The specimens were subjected to a cyclic fatigue test at the frequency of 2 Hz, considering hybrid strain–stress control. The applied loading semi-cycle was expressed as the value of absolute displacement end level Δ*l* = 0.35 mm (in each cycle). The unloading semi-cycle was equal to a nominal stress *σ_min_* = 0, resulting in a coefficient of asymmetry of the mixed cycle *R_εσ_* = 0. The experiment was performed using an MTS 809 A/T servohydraulic testing machine (MTS System, Eden Prairie, MN, USA). The loading process was controlled using an Epsilon 3448 extensometer (Epsilon Tech, Jackson, MS, USA) with an initial specimen gauge part *l*_0_ = 25 mm. To sum up the experimental procedure, the loading part of the cycle was controlled by a constant value of displacement (extensometer), while the unloading part of the cycle completed at close-to-zero tensile force values (controlled by the MTS load cell). This resulted in the strain value decreasing with each cycle, because of the increasing permanent elongation of the specimen.

**Figure 4:**
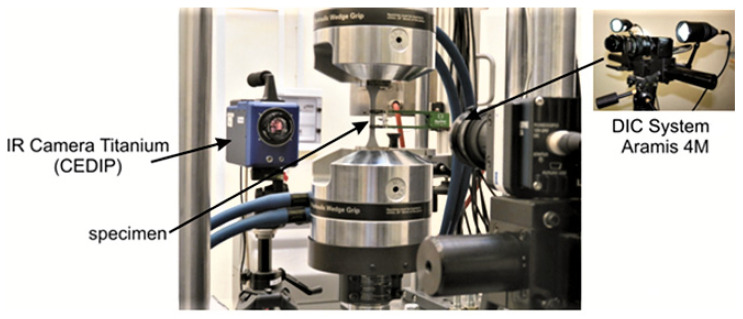
The measuring system.

In the course of the cyclic deformation process, the evolution of the temperature field on the surface of the tested specimen was measured using an IR thermographic system CEDIP Titanium (Cedip Infrared Systems, Croissy Beaubourg, France); the strain field on the opposite specimen surface was simultaneously recorded by means of a digital image correlation system Aramis 3D 4M (GOM, Braunschweig, Germany). Figure 4 shows the measuring system used during experimental procedures.

**Figure 5:**
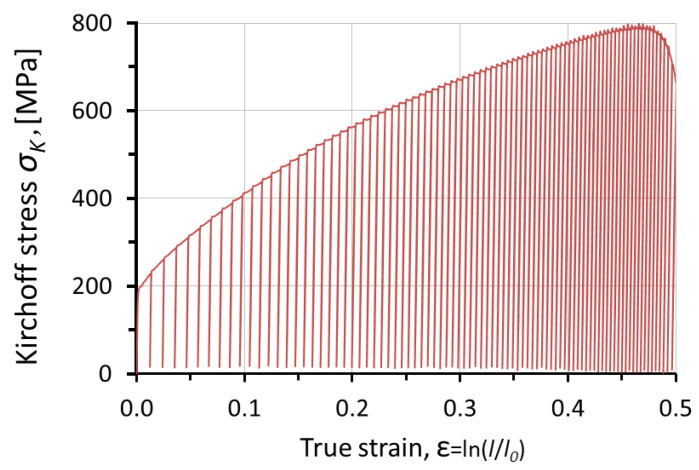
Kirchhoff stress–true strain dependency.

The data acquisition frequency of the measured fields was equal to 80 Hz. The force value *F*(*t*) and current length of the gauge part of the specimen *l*(*t*) were simultaneously recorded as a function of time *t*. The Kirchhoff stress,

(4) $$\sigma_{K}=\frac{l\left(t\right)}{l_{0}}\frac{F\left(t\right)}{A_{0}},$$

and true strain,

(5) $$\varepsilon =ln\frac{l\left(t\right)}{l_{0}},$$

were calculated, considering *l*_0_ as the initial length of the gauge part of the specimen, *A*_0_ as its cross-section, and *l*(*t*) as the current length of the gauge part. Figure 5 shows the results obtained during the low-cycle deformation process of the mixed cycle *R_εσ_* = 0.

**Figure 6:**
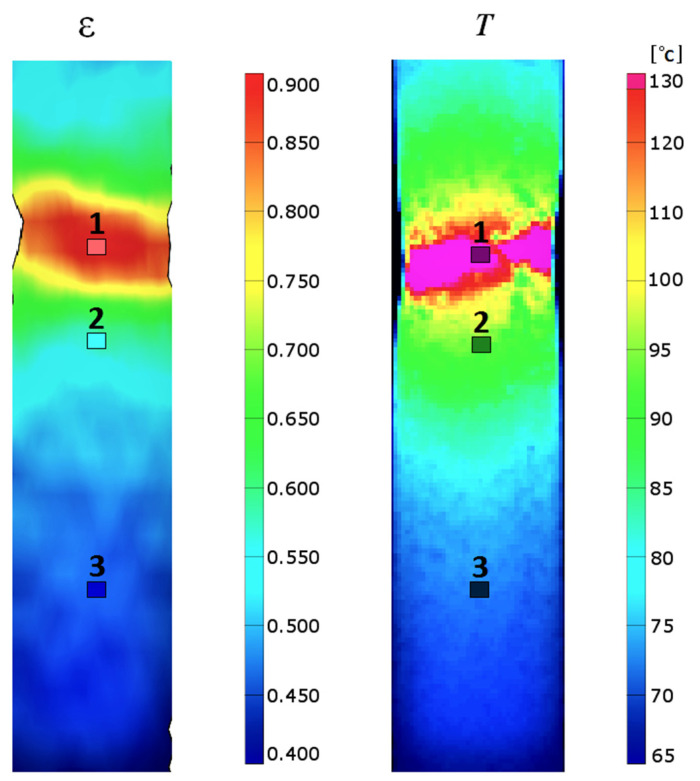
Strain and temperature fields just before the specimen fracture.

All measured quantities during the experimental procedure were recorded as a function of time or the number of cycles. This approach enabled determining the correlation between the recorded values. The temperature and strain fields during the experimental process were recorded and simultaneously calculated on the surface of the deformed specimens. The homogeneous microstructure of the specimens in the initial state led to the expectation that the plastic strain localization and fracture of the specimen would appear in the middle of its gauge part. Section 1 and Section 2 were selected as the areas in which the localization of plastic strain was expected. The localization of plastic strain is a result of the increase in strain rate of a certain area of the deformed specimen as compared to its other areas. The increased rate of deformation causes an increase in temperature. The deformation and temperature fields just before the specimen fracture are shown in Figure 6.

## 3. Results and Discussion

The main goal of the present work was to determine the energy storage rate in the area of plastic strain localization. Localization occurs in the final stage of the deformation process. Despite the uniaxial loading of the specimen, in the plastic strain localization area, the stress is not equal to *σ_K_*, but a three-dimensional, nonuniform stress state. Determining the increments of plastic work for the selected surface sections requires taking into account such a stress state.

**Figure 7:**
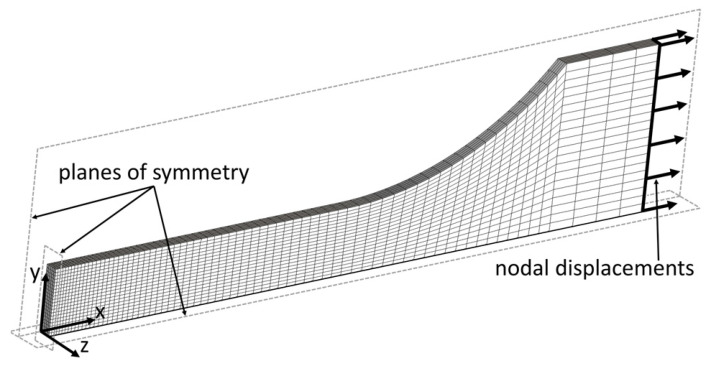
Finite element mesh and boundary conditions used for the numerical model.

The stress and strain tensors can be derived into their spherical and deviator components. It can be assumed that, in the area of plastic strain localization, the spherical component is negligibly small. Therefore, the distribution of the effective stress *σ_eq_* (equivalent stress) was acquired by the finite element method (FEM) using MSC.Marc software. The calculations were carried out for a three-dimensional model including eight-node, hexahedral finite elements. The geometric model was divided into 7400 finite elements densified in the *x*-axis direction, where the expected maximum strain was located (Figure 4). The symmetric boundary conditions were applied, assuming the displacements *u_x_* = 0, *u_y_* = 0, and *u_z_* = 0 for the nodes lying on the *y_z_*-plane, *x_z_*-plane, and *x_y_*-plane, respectively. In order to deform the model, nodal displacements in the direction of the positive *x*-axis values (*u_x_* = s) were used. To determine the true stress–strain curve of the 304L steel, a hybrid method (experimental–numerical) was used [26,27,28]. At first, the Kirchhoff stress–true strain curve obtained experimentally was used for numerical calculations (Figure 5), and then the force–displacement relationship obtained by means of calculations and experiment were compared. Due to the lack of convergence of force–displacement relationships, in subsequent iterations, the material stress–strain curve used in numerical calculations was changed. Changes to the true stress–strain curve were applied until a high compliance of the numerical and experimental tensile curves was obtained. The true curve was further used to define the plastic deformation behavior of 304L steel and values of equivalent stress (Huber–von Mises stress) *σ_eq_*. The values of equivalent stress and strain during the tension semi-cycle were obtained from a node located inside the center of the gauge length of the model, i.e., at the starting point of the coordinate system shown in Figure 7.

**Figure 8:**
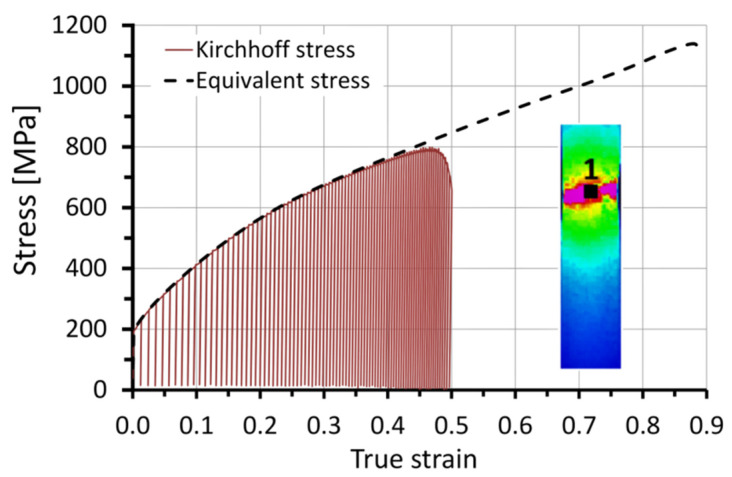
Kirchhoff and equivalent stress dependency, along with a thermal image of the tested specimen before fracture.

The dependences of Kirchhoff and equivalent stress on true strain are shown in Figure 8. The presented stress–strain dependency refers to quantities measured in the gauge part of the specimen during the experiment, as well as the results obtained from the numerical calculations using MSC.Marc software. Point 1 marked in the thermal image of the tested specimen corresponds to the area of specimen fracture. The difference between *σ_K_* stress and Huber–von Mises stress *σ_eq_* can be treated as an indicator of plastic strain localization (Figure 8).

**Figure 9:**
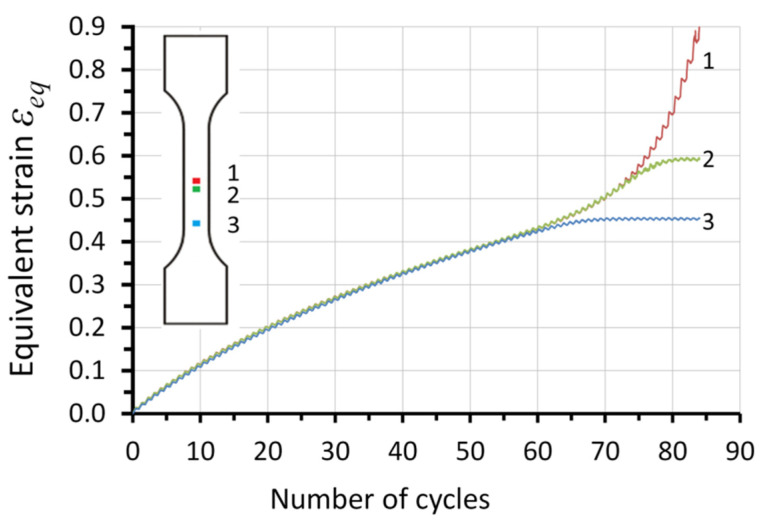
Equivalent strain–number of cycles dependency in the selected sections.

Before the equivalent strain reached a critical threshold, the specimen deformed uniformly. The strain in all considered sections of the specimen was equal to the true strain value measured in the gauge part. This was confirmed by the dependence of the strain of the three selected sections (1, 2, and 3) with reference to the number of cycles (Figure 9). The average strain value of a given section was calculated in terms of the recorded displacement field on the specimen surface by means of the digital image correlation system (Aramis 3D 4M). After the strain reached the critical threshold value *ε_eq_* = 0.40, the strain rate of particular sections began to differ. Differences between Kirchhoff and Huber–von Mises stress (Figure 8) occurred after reaching the strain value at which strain heterogeneity occurred, i.e., *ε* = *ε_eq_* = 0.40.

**Figure 10:**
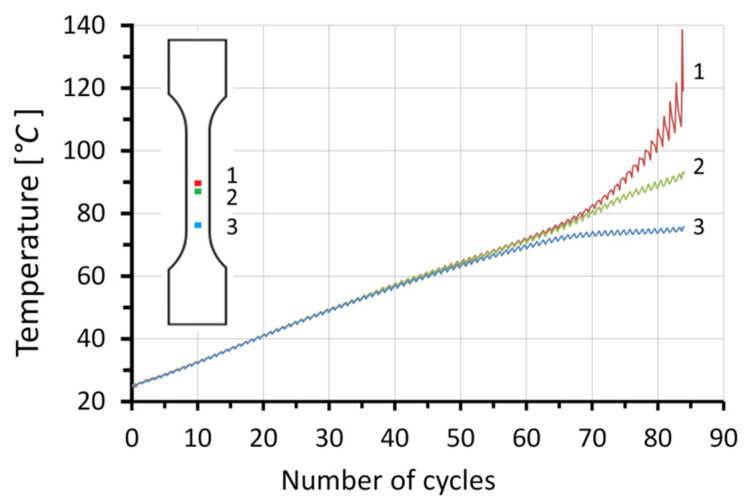
Temperature–number of cycles dependency in the selected sections.

During plastic deformation, part of the energy delivered to the specimen is dissipated in the form of heat. This causes the temperature growth of the specimen. The increase in temperature is proportional to the strain rate. If the strain rate field is uniform, the temperature increase of these areas over time is also uniform. The tested specimens deformed uniformly until the 55th cycle. Toward the end of the process, the individual sections of the specimen deformed at different strain rates, resulting in different temperature increases in particular sections (Figure 10).

### 3.1. Determination of Plastic Work Increments Considering Selected Sections of the Specimen

The presented energy storage rate is expressed in terms of the increments of energy dissipated Δ*q_d_*(*t,n*) and plastic work Δ*w_p_*(*t,n*). As a measure of the deformation time *t*, the number of cycles *N_f_* was used. Determination of Δ*w_p_*(*t,n*) and *q_d_*(*t,n*) for a particular specimen section *n* allowed the calculations of energy storage rate for the selected section. The increment of plastic work is expressed as follows:

(6) $$\Delta w_{p}=\frac{1}{\rho }\int_{\varepsilon_{p1}}^{\varepsilon_{p2}}\sigma_{eq}d\varepsilon_{p},$$

where *σ_eq_* is the Huber–von Mises stress, *ε_p_* is the plastic strain, and *ρ* is the mass density of the tested steel. By assuming a constant value of Young’s modulus during the experimental procedure, the value of local plastic strain can be obtained from the following equation:

(7) $$\varepsilon_{p}\left(t\right)=\varepsilon_{eq}\left(t\right)-\frac{\sigma_{eq}}{E},$$

where *E* is Young’s modulus. The increments of Δ*w_p_*(*t,n*) and *q_d_*(*t,n*) for selected sections were determined for deformation time intervals of Δ*t* = 0.25 s during subsequent cycles. The stress and strain changes are denoted by Δ*σ_eq_*(*t,n*) and Δ*ε_eq_*(*t,n*).

To avoid determining the mathematical form of the dependence Δ*σ_eq_*(*ε_eq_*), for successive changes in strain Δ*ε_eq_*(*t,n*), the average values of Huber–von Mises stress *σ_eq-av_* were obtained from the numerical calculations *σ_eq_*(*ε_eq_*) (Figure 8). Then, using Equation (7), Δ*ε_p_* was calculated. The plastic work increments at successive time intervals Δ*t* corresponding to successive cycles were determined using the following form of Equation (6):

(8) $$\Delta w_{p}=\frac{1}{\rho }\int_{\varepsilon_{p1}}^{\varepsilon_{p2}}\sigma_{eq}d\varepsilon_{p}\approx \frac{1}{\rho }\sigma_{eq-av}\Delta \varepsilon_{p}.$$

### 3.2. Determination of Dissipated Energy Increments as Heat in the Selected Specimen Sections

**Figure 11:**
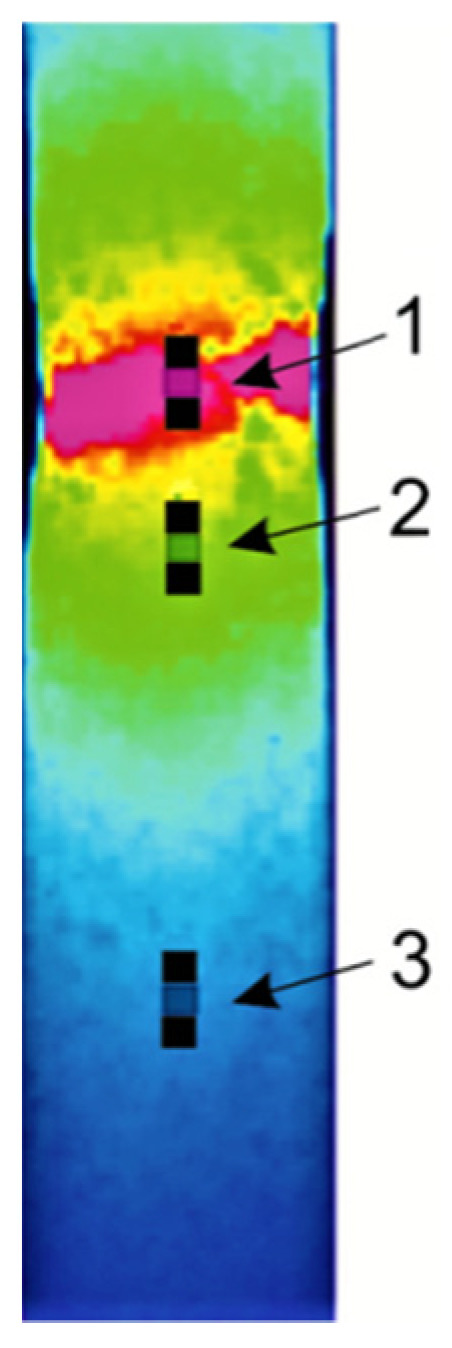
Neighboring areas *n_up_* and *n_d_* of sections 1, 2, and 3.

Part of the energy equal to the increment of mechanical energy expended on plastic deformation Δ*w_p_*(*t,n*) is dissipated in the form of heat *q_d_*(*t,n*). An increase in specimen temperature is caused by the described increment of heat and depends on the value of strain rate. The distribution of strain rate is not uniform in the area of plastic strain localization, as shown in Figure 6. The temperature field during the experimental procedure was recorded as a function of time by means of the IR thermography system. On the basis of the obtained experimental data, the average temperature *T_av_*(*t,n*) of each section was determined, for *n =* 1, 2, and 3. In the calculations of heat increment *q_d_*(*N_f_,n*) generated during the loading semi-cycle (Δ*t* = 0.25 s), considering the number of cycles *N_f_* in each of the selected sections, the following components were included:

- the heat increment Δ*q*_1_ causing the rise in temperature of a unit mass of the tested material by Δ*T* during the time interval Δ*t* = 0.25 s,

(9) $$\Delta q_{1}\left(N_{f},n\right)=c_{w}\times \Delta T\left(N_{f},n\right),$$

where *c_w_* is the specific heat of the tested steel, and *N_f_* is the number of cycles;

- the heat increment Δ*q*_2_ spent for compensation of the temperature drop due to the thermo-elastic effect,

(10) $$\Delta q_{2}\left(N_{f},n\right)=\frac{\alpha T_{av}\left(N_{f},n\right)\Delta \sigma_{eq}(N_{f},n)}{\rho },$$

considering *α* as the coefficient of linear thermal expansion, *T_av_*(*N_f_,n*) as the average value of absolute temperature in the selected section, and Δ*σ_eq_*(*N_f_,n*) as the strain increment during the given cycle;

- the heat transfer, according to Fourier’s law, between given area *n* and its neighbors *n_up_* and *n_d_* (Figure 11), expressed as

(11) $$\Delta q_{3}=\frac{S_{n/n_{up}}\left(N_{f},n\right)\cdot \lambda \cdot \Delta t}{\rho_{0}\cdot V_{0}}\cdot \left[\frac{T\left(N_{f},n\right)-T\left(N_{f},n_{up}\right)}{d}\right]+\frac{S_{n/n_{d}}\left(N_{f},n\right)\cdot \lambda \cdot \Delta t}{\rho_{0}\cdot V_{0}}\;\cdot \left[\frac{T\left(N_{f},n\right)-T\left(N_{f},n_{d}\right)}{d}\right],$$

where *λ* is the thermal conductivity, *V*_0_ is the volume of the considered section. $S_{n/n_{up}}\left(N_{f},n\right)$ and $S_{n/n_{d}}\left(N_{f},n\right)$ are the cross-sectional areas between neighboring sections *n*/*n_up_* and *n*/*n_d_*, respectively, and *d* = 1 mm is the distance between the centers of neighboring areas.

The addition of all preceding heat components resulted in heat increments Δ*q_d_* for selected specimen sections 1, 2, and 3 during the low-cycle fatigue test (Equation (12)).

(12) $$\begin{array}{ll} \Delta q_{d}= & \Delta q_{1}\left(N_{f},n\right)+\Delta q_{2}\left(N_{f},n\right)+\Delta q_{3}\left(N_{f},n\right)=c_{w}\cdot \Delta T\left(N_{f},n\right)+\\ & \frac{\alpha T_{av}\left(N_{f},n\right)\Delta \sigma_{eq}(N_{f},n)}{\rho }+\frac{S_{n/n_{up}}\left(N_{f},n\right)\cdot \lambda \cdot \Delta t}{\rho_{0}\cdot V_{0}}\\ & \cdot \left[\frac{T\left(N_{f},n\right)-T\left(N_{f},n_{up}\right)}{d}\right]+\frac{S_{n/n_{d}}\left(N_{f},n\right)\cdot \lambda \cdot \Delta t}{\rho_{0}\cdot V_{0}}\\ & \cdot \left[\frac{T\left(N_{f},n\right)-T\left(N_{f},n_{d}\right)}{d}\right]. \end{array}$$

### 3.3. Calculation of the Energy Storage Rate for the Selected Specimen Sections

**Figure 12:**
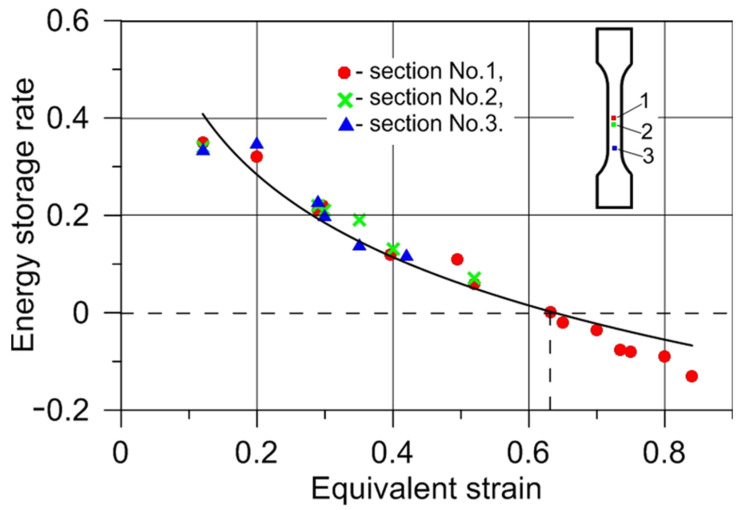
Energy storage rate–equivalent strain dependency during low-cycle fatigue of austenitic stainless steel.

**Figure 13:**
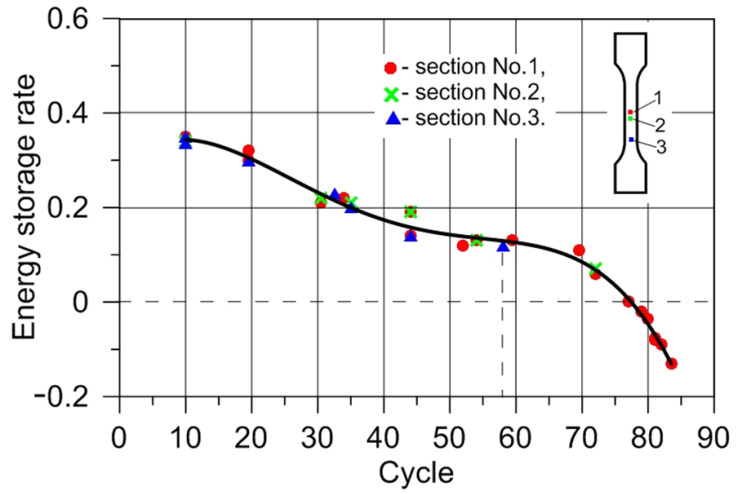
Energy storage rate–number of cycles dependency during low-cycle fatigue of austenitic stainless steel.

The dissipated energy increments in the form of heat in the selected specimen sections were determined using the local form of the heat equation (Equation (12)). Plastic work increments were obtained with the use of Equation (8). Using Equation (3) for each of the considered sections (1, 2, and 3), the energy storage rate *Z* was calculated. The heat transfer in a perpendicular direction to the specimen axis compared to the heat transport to the grips was assumed negligible. The density of the tested steel during its plastic deformation was considered constant. The recorded course of temperature and the strain fields on the specimen surface were used for the determination of average temperature and equivalent stress increments for the selected specimen section, considering subsequent time intervals. The correlation of the energy storage rate with the equivalent strain *ε_eq_* and the number cycles *N_f_* for the selected sections of the tested specimens is presented in Figure 12 and Figure 13, respectively.

Figure 12 shows that the energy storage rate for all considered sections decreased with the increasing value of strain. With the development of plastic strain localization, its area dimensions decreased; section 3 stopped deforming, whereas section 1 continued deforming until the specimen cracked (Figure 9). The tested specimens fractured in section 1, at *ε_eq_* = 0.9, corresponding to 85 cycles. The crack occurred in the area where the material subjected to low-cycle fatigue lost its energy storage ability (energy storage rate *Z* = 0). Before the fracture, the stored energy was released as heat (energy storage rate *Z* < 0). The results of the presented study show that the specimen subjected to low-cycle loading, similarly to the specimen subjected to monotonic uniaxial tension [23], lost its energy storage ability in the region of plastic strain localization, and the energy storage rate dropped to zero (Figure 13). The obtained results strengthen the hypothesis that the plastic instability criterion can be determined on the basis of close-to-zero values of the energy storage ability. A state of plastic instability may be reached by a polycrystalline material subjected to any type of loading. The occurrence of a zero value of the energy storage rate, in specimens subjected to low-cycle fatigue, may be treated as an indicator of the fatigue fracture initiation. Before the deformed specimen failure, the energy storage rate dropped to a negative value (*Z* < 0). This means that, after reaching the state of plastic instability, the energy stored in the previous stages of the deformation process was released in the form of heat. The release of the stored energy may be connected to the evolution of damage mechanisms.

## 4. Conclusions

Using IR thermography and a DIC system, the energy storage process in 304L austenitic stainless steel subjected to low-cycle fatigue was investigated. According to the strain field measurement, the area of plastic strain localization was specified. Calculation of the energy storage rate required knowledge of the plastic work and increments of energy dissipated in the form of heat in subsequent load cycles. Three sections of the tested specimen were selected for determination of the energy storage rate as a function of the number of cycles. The three-dimensional, nonuniform stress state in the area of plastic strain localization was used during calculations of the plastic work increments. Dissipated energy in the form of heat considering the selected sections was determined using the local form of the heat equation and the measured temperature fields. On the basis of the obtained results, the energy storage rate *Z* dependency on the strain and the number of cycles was calculated. It was shown that, in the austenitic stainless steel subjected to low-cycle loading, before the specimen fracture, the energy stored during the deformation process was released as heat (energy storage rate *Z* < 0). The material reached a state in which it lost its ability to store energy. Zero and negative values of the energy storage rate can be regarded as a plastic instability criterion.

During a low-cycle fatigue process of the tested material, a zero value of the energy storage rate could be considered as an indicator of a fatigue crack initiation. It is worth emphasizing that this indicator resulted from a thermodynamic analysis of the low-cycle fatigue process. The release of stored energy may be connected to the evolution of damage mechanisms. Identification of these mechanisms requires reference of the negative values of energy storage rate to the changes in microstructure of the tested material leading to fatigue crack initiation in the specimen. This will be the subject of further research.

## Figures and Tables

**Table 1:** Chemical composition of 304L austenitic stainless steel.

Chemical Composition (%)												
C	Mn	Si	P	S	Cr	Ni	W	Mo	Cu	V	Ti	Fe
0.05	1.35	1.0	0.016	0.008	18.58	17.3	0.025	0.02	0.04	0.03	0.013	bal.

## Data Availability

Data sharing not applicable.
